# The Validity of MotionSense HRV in Estimating Sedentary Behavior and Physical Activity under Free-Living and Simulated Activity Settings

**DOI:** 10.3390/s21041411

**Published:** 2021-02-18

**Authors:** Sunku Kwon, Neng Wan, Ryan D. Burns, Timothy A. Brusseau, Youngwon Kim, Santosh Kumar, Emre Ertin, David W. Wetter, Cho Y. Lam, Ming Wen, Wonwoo Byun

**Affiliations:** 1 Department of Health and Kinesiology, University of Utah, Salt Lake City, UT 84112, USA; sunku.kwon@utah.edu (S.K.); ryan.d.burns@utah.edu (R.D.B.); tim.brusseau@utah.edu (T.A.B.); 2 Department of Geography, University of Utah, Salt Lake City, UT 84112, USA; neng.wan@utah.edu; 3 School of Public Health, The University of Hong Kong Li Ka Shing Faculty of Medicine, Hong Kong; youngwon.kim@hku.hk; 4 MRC Epidemiology Unit, University of Cambridge School of Clinical Medicine, Cambridge CB2 0SL, UK; 5 Department of Computer Science, University of Memphis, Memphis, TN 38152, USA; skumar4@memphis.edu; 6 Department of Electrical and Computer Engineering, The Ohio State University, Columbus, OH 43210, USA; ertin.1@osu.edu; 7 Department of Population Health Sciences and Huntsman Cancer Institute, University of Utah, Salt Lake City, UT 84132, USA; David.Wetter@hci.utah.edu (D.W.W.); Cho.Lam@hci.utah.edu (C.Y.L.); 8 Department of Sociology, University of Utah, Salt Lake City, UT 84112, USA; ming.wen@soc.utah.edu

**Keywords:** mobile health, sedentary behavior, physical activity, validity, MotionSense HRV, accelerometer

## Abstract

MotionSense HRV is a wrist-worn accelerometery-based sensor that is paired with a smartphone and is thus capable of measuring the intensity, duration, and frequency of physical activity (PA). However, little information is available on the validity of the MotionSense HRV. Therefore, the purpose of this study was to assess the concurrent validity of the MotionSense HRV in estimating sedentary behavior (SED) and PA. A total of 20 healthy adults (age: 32.5 ± 15.1 years) wore the MotionSense HRV and ActiGraph GT9X accelerometer (GT9X) on their non-dominant wrist for seven consecutive days during free-living conditions. Raw acceleration data from the devices were summarized into average time (min/day) spent in SED and moderate-to-vigorous PA (MVPA). Additionally, using the Cosemed K5 indirect calorimetry system (K5) as a criterion measure, the validity of the MotionSense HRV was examined in simulated free-living conditions. Pearson correlations, mean absolute percent errors (MAPE), Bland–Altman (BA) plots, and equivalence tests were used to examine the validity of the MotionSense HRV against criterion measures. The correlations between the MotionSense HRV and GT9X were high and the MAPE were low for both the SED (r = 0.99, MAPE = 2.4%) and MVPA (r = 0.97, MAPE = 9.1%) estimates under free-living conditions. BA plots illustrated that there was no systematic bias between the MotionSense HRV and criterion measures. The estimates of SED and MVPA from the MotionSense HRV were significantly equivalent to those from the GT9X; the equivalence zones were set at 16.5% for SED and 29% for MVPA. The estimates of SED and PA from the MotionSense HRV were less comparable when compared with those from the K5. The MotionSense HRV yielded comparable estimates for SED and PA when compared with the GT9X accelerometer under free-living conditions. We confirmed the promising application of the MotionSense HRV for monitoring PA patterns for practical and research purposes.

## 1. Introduction

With advances in mobile technology, the field of mobile health (mHealth) has attracted the attention of healthcare providers interested in efficient patient care. The World Health Organization defines mHealth as the practice of public health and medicine supported by mobile devices such as smartphones. mHealth embodies a healthcare system that capitalizes on mobile devices’ voice and text messaging service, wireless telecommunications (e.g., LTE network), Bluetooth® technology, and global positioning system (GPS) [1]. mHealth technology allows for more people to connect with innovative health care services, including health care management, health care information on demand, and the real-time monitoring of behavior and chronic conditions. For instance, mHealth technology can efficiently and quickly support healthcare providers’ remote clinical care through the periodical or real-time monitoring of patients’ physiological factors (e.g., heart rate) and health-related behaviors (e.g., physical activity (PA)) [2,3,4]. Thus, advances in mHealth technology are expected to significantly improve the clinical and wellness care for various populations by reducing the cost and burden of the evaluation of risk factors of potential chronic disorders.

The National Center of Excellence for Mobile Sensor Data-to-Knowledge (MD2K) is a part of the Big Data-to-Knowledge program funded by the National Institutes of Health. An overarching goal of the MD2K is to use mobile sensor technologies to detect and predict behavioral, psychological, and environmental risk factors of specific diseases [5,6]. The ability to detect risk factors and prevent the emergence of adverse clinical events is an essential strategy in preventive medicine and can help to reduce health care costs [5,6,7]. Recently, the MD2K developed an innovative multi-sensor approach named puffMaker to objectively track smoking episodes using two wearable sensors called AutoSense and MotionSense [8]. AutoSense is a chest-worn sensor suite that could measure breathing patterns [9,10]. MotionSense is a wrist-worn inertial sensor equipped with a 3-axis accelerometer and a 3-axis gyroscope for detecting accelerations and movements of the arms [11]. The acceleration and angular motion data collected at wrists can be translated into the intensity and amount of wrist movements. In fact, the use of wrist-worn accelerometers for assessing PA is now widespread [12,13]. This suggests that the MotionSense has tremendous potential to be a device for monitoring PA if its concurrent validity is confirmed. 

Recently, the MD2K team released MotionSense HRV, an upgraded version of the MotionSense that additionally includes a multispectral photoplethysmography (PPG) sensor [14,15]. With the additional sensors, the MotionSense HRV is capable of measuring various physiological and behavioral variables, including smoking events, heart rates, eating episodes, cocaine use, and brushing teeth [5,8,14,15]. The MotionSense HRV is paired with a smartphone via Bluetooth Low Energy. It transfers raw data to the mCerbrum mobile application, an open-source mobile software platform developed by MD2K that stores and processes the raw data to make real-time inferences about the user’s state using machine learning models that can be used to trigger interventions on the mobile phone [16] The mCerebrum application allows the MotionSense HRV to collect data at a high sampling frequency (i.e., ≥70 million samples/day) and compute the data in real time. The use of the MotionSense HRV and mCerebrum enables researchers to monitor users’ physiological (e.g., heart rate) and behavioral changes (e.g., levels of PA) [5,17]. 

PA plays a critical role in reducing the risks of obesity, diabetes, cancer, and cardiovascular diseases [18], thus, the study of PA is becoming a top-priority area in public health and clinical research. However, due to the complexity and intermittent patterns of PA performed in daily life, accurately assessing an individual’s PA level through subjective measures (i.e., self-reported questionnaires) is particularly challenging as the methods are vulnerable to recall and social-desirable biases [19]. Accelerometry-based activity monitors have been widely accepted as devices that can objectively assess an individual’s PA levels in free-living conditions [20]. An accelerometer is a sensor that measures the acceleration of an object’s movement along its reference axes, thus the data it captures reflects the intensity and frequency of movement [21]. MotionSense HRV’s built-in accelerometer records accelerations relative to the input force oriented to multi-orthogonal planes of motion within distinct time intervals (i.e., epochs). Thus, it can be used to estimate the intensity, duration, and frequency of bodily movements. This suggests that the MotionSense HRV can potentially be utilized to monitor users’ PA and other behavioral and clinical measures concurrently. This device can also provide such information to healthcare providers for enhanced counseling. Despite the great potential of MotionSense HRV in PA monitoring, the concurrent validity of MotionSense HRV in estimating a user’s PA has not been systematically evaluated against previously established criterion measures. Establishing the validity of PA estimates is an essential step toward ensuring that MotionSense HRV can be used to monitor users’ PA patterns. Therefore, the purpose of this study was to assess the validity of MotionSense HRV for estimating time spent in sedentary behavior and PA against indirect calorimetry and a research-grade accelerometer. 

## 2. Materials and Methods

### 2.1. Design

This cross-sectional study was conducted in both free-living and laboratory-based simulated free-living settings. Sedentary behavior commonly refers to activities that require very low energy expenditure (≤1.5 metabolic equivalents), and PA is defined as any bodily movement produced by the contraction of skeletal muscles that substantially increases energy expenditure (>1.5 metabolic equivalents). The estimated time spent in sedentary behavior and physical activities from MotionSense HRV was compared to two criterion measures: (1) the ActiGraph GT9X accelerometer for a free-living condition; and (2) the Cosmed K5 portable indirect calorimetry system for simulated free-living conditions.

### 2.2. Participants

A convenient sample of 20 Pacific Islanders (10 Tongan Americans and 10 Samoan Americans; age range: 18–65 years) participated in this study. Pacific Islanders are a population group that suffers from disproportionately high burdens of obesity and its related health consequences, such as cardiovascular diseases, diabetes, and cancers [22,23,24,25]. Considering the potential utilization of the MotionSense HRV for obesity and cancer patients, we chose Pacific Islanders as our study participants. Participants were recruited via word of mouth, email, and flyers at the National Tongan American Society and the Queen Center in Salt Lake City in Utah. The directors and community leaders at those centers collaborated on recruiting the eligible participants and the follow-up with the participants of this study. Participants who were able to (1) participate in PA without any functional impairment, (2) use a smartphone, and (3) communicate in English (speak, read, and write) were eligible to participate in this study. Exclusion criteria included the following: (1) anyone with an implanted cardiac device, such as a pacemaker, (2) those who were physically unable to wear equipment and use a smartphone; (3) individuals who were pregnant or lactating; and (4) people with any unstable medical or psychiatric problems. The study protocols were approved by the University of Utah Institutional Review Board (IRB approval number: 00109145). 

### 2.3. Instruments

#### 2.3.1. MotionSense HRV

The MotionSense HRV is a custom wrist-worn sensor developed by the MD2K. The MotionSense HRV includes a tri-axial accelerometer and tri-axial gyroscope, a multispectral (red-, green-, infrared-light emitting diodes) PPG sensor, and a microcontroller [15]. The MotionSense HRV can track (1) hand gestures and arm movements through accelerometers and gyroscopes and (2) interbeat intervals from optical sensors for calculating heart rate variability [5]. Recently, the arm movement tracking of the MotionSense HRV was used for the puffMarker model, which can detect the timing of a lapse in smoking cessation by tracking arm movements [8]. Additionally, the MotionSense HRV could potentially be used to assess stress (i.e., cStress model) by integrating the measures of accelerations, interbeat intervals, and heart rate variability [26]. The data collected by the MotionSense HRV are transmitted to a smartphone in real time using the built-in microcontroller [15]. Given the evidence, the measures of time spent in sedentary behavior and PA as well as other health information by the MotionSense HRV in everyday life can potentially provide several benefits: (1) the periodical or real-time detection of health-related risk factors such as excessive sedentary behavior; (2) the convenient self-monitoring of various health-related behaviors with a wrist-worn sensor and mobile application; (3) the real-time sharing of various types of health information with healthcare providers for efficient counseling; (4) the delivery of interventions for smoking cessation by identifying smoking patterns. 

#### 2.3.2. ActiGraph GT9X Accelerometer

The ActiGraph GT9X Link (GT9X; ActiGraph Corp, Pensacola, FL, ISA) was used as another criterion method for evaluating the validity of the MotionSense HRV in estimating time spent in sedentary behavior and PA under free-living conditions. ActiGraph is the leading manufacturer of research-grade wearable activity monitors; ActiGraph accelerometers are most commonly used to examine sedentary behavior and PA in research [27,28,29,30,31,32]. The GT9X is the latest generation of research-grade accelerometer produced by ActiGraph. The GT9X is a small and light (3.5 × 3.5 × 1 cm; 14 g) device that can be worn on the wrist or at the waist using a manufacturer-provided wrist-strap or belt clip and features a tri-axial accelerometer at a dynamic range ± 8 g. The GT9X records vertical, anteroposterior, and mediolateral accelerations at a user-selected sampling rate (30–100 Hz). This device can be used to estimate a user’s PA intensity, activity and sedentary bouts, and steps taken at a user-selected epoch length (1–60 s) [27,31]. Previous studies have determined the validity of GT9X in estimating PA energy expenditure [27,33] and activity intensities [12] compared to an indirect calorimetry method, as well as wear-time detection compared to direct observation and self-reported methods [34]. Additionally, the reliability of the ActiGraph accelerometer for measuring sedentary behavior and PA under free-living conditions was confirmed in a previous study (standard error of the measurement < 11.2%) [35]. Accordingly, GT9X was previously used as a criterion measure to investigate the validity of consumer-based activity monitors in estimating PA in free-living conditions [36,37]. For that reason, the current study chose GT9X as a criterion measure to examine the validity of the MotionSense HRV in estimating time spent in sedentary behavior and PA during free-living conditions. 

#### 2.3.3. Indirect Calorimetry

The Cosmed K5 portable indirect calorimetry system (COSMED, Rome, Italy; K5) was used as a criterion measurement of PA during a simulated free-living session. Indirect calorimetry is a non-invasive technique that measures respiratory gas exchange to calculate energy expenditure. More specifically, indirect calorimetry measures inspired and expired gas flows, volumes, and concentrations of oxygen (O_2_) and carbon dioxide (CO_2_). The expired concentrations of O_2_ and CO_2_ can be used to calculate oxygen uptake (VO_2_) and carbon dioxide production (VCO_2_), which can, in turn, be used to estimate energy expenditure [38]. Indirect calorimetry is commonly used to calculate respiratory quotient (CO_2_ production/O_2_ uptake) and resting energy expenditure, as well as to determine caloric needs in research [39,40,41]. The K5, our criterion measure, is a portable indirect calorimetry system (174 × 111 × 64 mm and 900 g, including battery and O_2_ sensor) that is worn on the back with a harness [42]. 

Generally, portable indirect calorimetry systems are used for measuring maximal oxygen uptake (VO_2max_) in specific exercise conditions, validating and calibrating accelerometer-based physical activity monitors in the laboratory and free-living conditions (up to 6 continuous hours), and calculating physical activity energy expenditure in free-living conditions [42]. Previous studies have determined the validity and the reliability of the K5. They showed that the K5′s measurements of metabolic parameters (pulmonary ventilation, oxygen consumption, and carbon dioxide production) were comparable to the measurements from the breath-by-breath method of the traditional metabolic cart (Vyntus CPX; Jaeger-CareFusion, Höchberg, Germany), the automated breathing metabolic simulator (VacuMed automated system; VacuMed, USA), and the Douglas Bag Method [42,43,44]. Additionally, a recent study reported very high intra- and inter-device reliability of the K5 in measuring pulmonary ventilation, oxygen consumption, and carbon dioxide production (r > 0.99) [44].

Moreover, the K5 has been utilized as a criterion measurement to examine the validity of wearable activity monitors in estimating activity intensities [45]. The main unit of the K5 communicates with the OMNIA Metabolic software (COSMED, Rome, Italy) on a computer via Bluetooth and is capable of storing up to 2,048,000 breaths. Prior to the simulated free-living session, the K5 was calibrated using the manufacturer’s recommendations: (1) a flow meter calibration using a 3-L syringe, (2) a scrubber calibration that zeros the carbon dioxide (CO_2_) analyzer, (3) a reference gas calibration using a reference gas (16% O_2_, 5% CO_2_, 79% nitrogen). During the simulated free-living session, we used the Breath-by-Breath test mode (measures pulmonary ventilation and gas exchange) to measure oxygen uptake VO_2_ (mL·min^−1^) values [42]. 

### 2.4. Procedures

Participants visited the University of Utah PA research laboratory on two separate occasions (1st visits: free-living session; 2nd visits: simulated free-living session). Upon arrival at the PA lab for the 1st visit, the participants completed informed consent and a pre-enrollment survey, which gathers sociodemographic information. Research staff measured the anthropometric characteristics of each participant using a stadiometer (ShorrBoard^®^, Olney, MD, USA) for height (cm), an electric body-scale (Seca 869, Hamburg, Germany) for weight (kg), and a tape measure (Baseline^®^ Evaluation Instruments, White Plains, NY, USA) for waist circumference. For the anthropometric measure, the participants were asked to wear minimal clothing and take off their shoes. Body mass index (BMI, kg/m^2^) was calculated based on the measured height and weight. Research staff measured all the anthropometric characteristics three times to avoid any measurement error. After the anthropometric measures, participants were fitted with an MotionSense HRV device on their non-dominant wrist. Then research staff paired the device with a smartphone and the mCelebrum mobile application, where raw acceleration data from the sensor were transferred and stored. The sampling rate of the MotionSense HRV was 25 Hz, which is the default configuration. After fitting the MotionSense HRV, a GT9X device was initialized with a sampling rate of 100 Hz using ActiLife 6 software (ActiGraph, Pensacola, FL, USA). The GT9X device was fitted on the participant’s non-dominant wrist using a manufacturer-provided wrist-strap. The GT9X was secured midway between the radial and the ulnar styloid processes [12]. Each participant was instructed on how to simultaneously wear MotionSense HRV and GT9X devices for the next 7 days during all waking hours except while performing aquatic activities (e.g., bathing or swimming) and charging the devices. Additionally, the participants were instructed to record (document) their daily sleep times and any non-wear time during waking hours on the sleep and non-wear time log.

Once the participants returned to the PA lab after the free-living condition, they first asked to complete the post-enrollment survey, which includes 12 questions about usability (e.g., the degree of the burden to carry devices and a smartphone, privacy issues, and troubleshooting experiences) of the MotionSense HRV during the free-living session. Then, participants were explained on the activity protocol for the simulated free-living session. During this session, each participant performed the activity protocol for 62 minutes in the gym. In the activity protocol, a total of 12 activities were categorized into three intensity categories (i.e., sedentary behavior, light PA, and moderate-to-vigorous PA) according to the PA compendium [46]. Each activity was selected to simulate activities that are commonly performed in free-living conditions (Table 1). Following the explanation about the activity protocol, the participant was fitted with the K5 indirect calorimetry with a face mask. Additionally, the participant wore the MotionSense HRV and GT9X on their non-dominant wrist. Participants performed each activity for 5 minutes and took a 1-minute break during the transition between each intensity category. Upon completing the entire activity protocol, data collected from the MotionSense HRV, GT9X, and K5 were immediately downloaded and securely stored for statistical analyses. 

### 2.5. Data Processing

#### 2.5.1. Activity Monitors

Data from the GT9X and MotionSense HRV were downloaded and saved as their raw data format using the ActiLife 6 software and the mCerebrum mobile application, respectively, and both types were converted into “.csv” files for further analyses. The R-package (http://cran.r-project.org (accessed on 15 January 2021)) designed for reducing multiday raw acceleration data called the GGIR package (version 1.10-10) [47] was used to calculate the amount of time spent in sedentary behavior and PA based on the intensity-specific milli-g cut-points derived from previously validated regression equations [48]. More specifically, the GGIR package calibrates the raw tri-axial acceleration data and converts it to the Euclidean norm minus one (ENMO;$\;\sqrt{x^{2}+y^{2}+z^{2}}-1g$). The ENMO indicates the value of gravity with negative values rounded to zero [49]. The ENMO values were categorized into different activity levels per one-second by applying the intensity thresholds for ENMO derived by Hildebrand et al [48,50]. Following the activity classification, the processed GT9X and MotionSense HRV data were merged and aligned into a single dataset. The single activity monitor dataset was collapsed into a 60-s epoch data for excluding the non-wear and sleep time. Non-wear time and sleep time were defined and excluded in the ActiLife software using Choi’s Algorithm [51] and self-reported activity/sleep log from each participant. Choi’s Algorithm detects the non-wear time if the accelerometer detects consecutive zero counts within a 90-minute window [51]. Additionally, Choi’s Algorithm regards nonzero counts of up to 2 minutes within 30-minute rolling windows of zero counts as artifactual accelerometer movements (e.g., the device is accidentally moved on a bedside table) during non-wear periods [51]. In addition, given that MotionSense HRV’s acceleration data could not be collected at the disconnecting moments with the smartphone, the times not recorded MotionSense HRV’s data were considered as invalid times in this study. We only included the data with valid time during waking hours of each day for statistical analyses.

#### 2.5.2. Indirect Calorimetry 

Breath-by-breath data from the K5 were exported in a “.csv” format by a 10-s epoch. The K5 data were collapsed into a 60-s interval and calculated to the metabolic equivalence of task (MET; 1 MET = 3.5 ml/kg/min) using measured VO_2_ (ml/min) and the body weight (kg) of each participant. Then, the calculated MET were classified into different activity levels (≤1.5 MET = sedentary behavior, 1.6 − 2.9 MET = Light PA, 3.0 − 5.9 MET = Moderate PA, ≥6.0 MET = Vigorous PA). Following the classification, the processed K5 data was merged with a single activity monitor dataset for statistical analyses.

### 2.6. Statistical Analysis

Descriptive analyses were conducted to summarize the sociodemographic and anthropometric characteristics of the participants. The normality of data was confirmed using the Shapiro–Wilk test. Thus, all statistical analyses were performed using parametric statistics. Pearson correlation coefficients were used to determine the relationship between estimates from the MotionSense HRV and those from the GT9X or the K5 indirect calorimetry. Measurement errors of the MotionSense HRV in comparison with the criterion measures were calculated based on mean absolute percent errors. Bland–Altman plots illustrated the agreement and systematic biases in the estimates of sedentary behavior and PA between the MotionSense HRV and criterion measures. Equivalence testing was performed to determine whether sedentary behavior and PA estimates from the MotionSense HRV are equivalent to those from the GT9X or the K5 indirect calorimetry [52]. The 90% confidence interval (CI) of the estimates from the MotionSense HRV were compared with the EZ from the K5 and the GT9X. Since there is no evidence of a universally acceptable EZ range, this study established a minimum EZ for the K5 and GT9X measures that include 90% CI of the MotionSense HRV estimates. Data were analyzed using the Stata 14.2 software and SAS 9.4 software (SAS Institute, Cary, NC, USA), and statistical significance was set at *p* < 0.05.

## 3. Results

The participant characteristics are presented in Table 2. The mean age of participants was 32.5 ± 15.1 years. Participants had an average weight of 90.1 ± 12.5 kg, with a BMI of 30.5 ± 4.0 kg/m^2^. Moreover, the results of the post-enrollment survey revealed that participants were generally acceptive of the smartphone and mCerebrum software and had little concerns over data privacy issues. The majority of the participants (i.e., 75%) were willing to use the system for longer-term measurements (Table S1).

**Figure 1:**
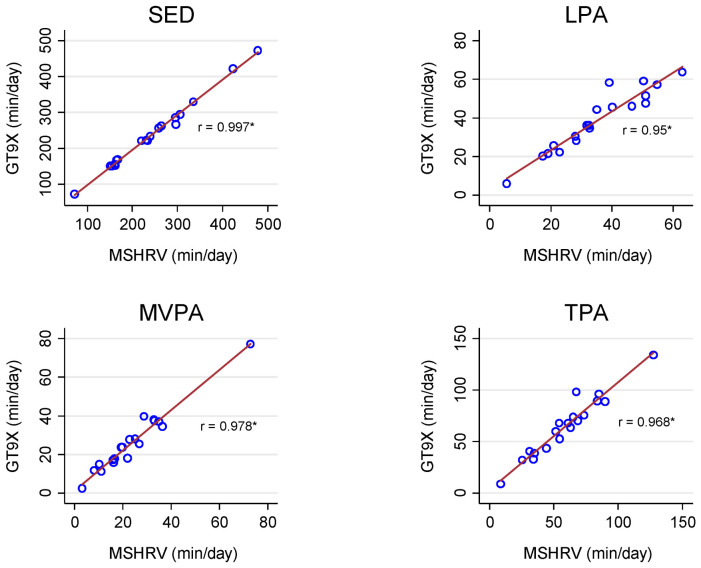
Pearson Correlations (r) for sedentary behavior and physical activity (PA) estimates between the GT9X and MotionSense HRV (MSHRV) under free-living conditions. SED: sedentary behavior; LPA: light PA; MVPA: moderate-to-vigorous PA; TPA: total PA; * *p* < 0.05.

**Figure 2:**
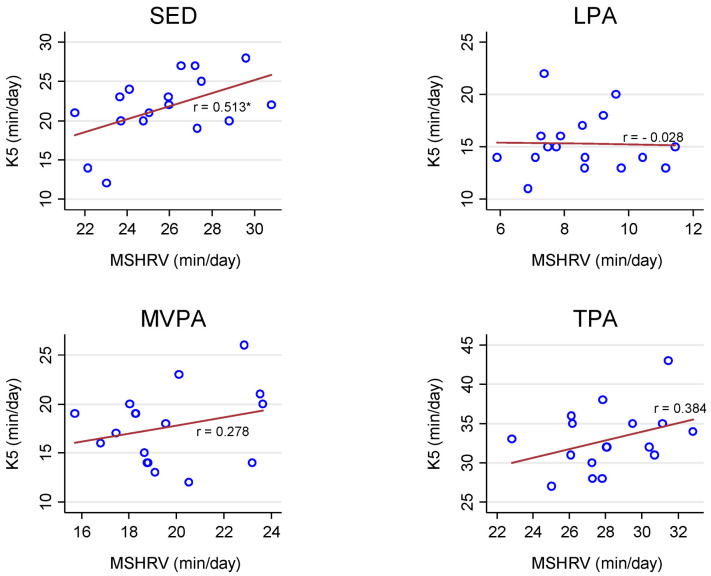
Pearson Correlations (r) for sedentary behavior and physical activity (PA) estimates between the Cosmed K5 (K5) and MotionSense HRV (MSHRV) under simulated free-living conditions. SED: sedentary behavior; LPA: light PA; MVPA: moderate-to-vigorous PA; TPA: total PA; * *p* < 0.05.

We observed very strong correlations for sedentary behavior and PA estimates between the GT9X and MotionSense HRV (range: r = 0.95 to 0.99, *p* < 0.01) under free-living conditions (Figure 1). In the simulated free-living conditions, the correlations between the K5 and MotionSense HRV were moderate for sedentary behavior (r = 0.51, *p* = 0.04) and total PA (r = 0.38, *p* = 0.13), weak for moderate-to-vigorous PA (r = 0.28, *p* = 0.28), and very weak for light PA (r = −0.03, *p* = 0.91; Figure 2). 

Overall, the mean differences in activity estimates across varying intensities were relatively small between the GT9X and MotionSense HRV (mean difference range: −5.7–5.7 min/day; mean absolute present error range: 2.4–9.1%) in the free-living conditions (Table 3). In the simulated free-living conditions, the mean differences in sedentary behavior and PA estimates between the Cosmed K5 and MotionSense HRV ranged from −4.1 min to 6.8 min; the mean absolute present error of MotionSense HRV in estimating sedentary behavior and PA ranged from 12.3% to 44.2% (Table 4). 

**Figure 3:**
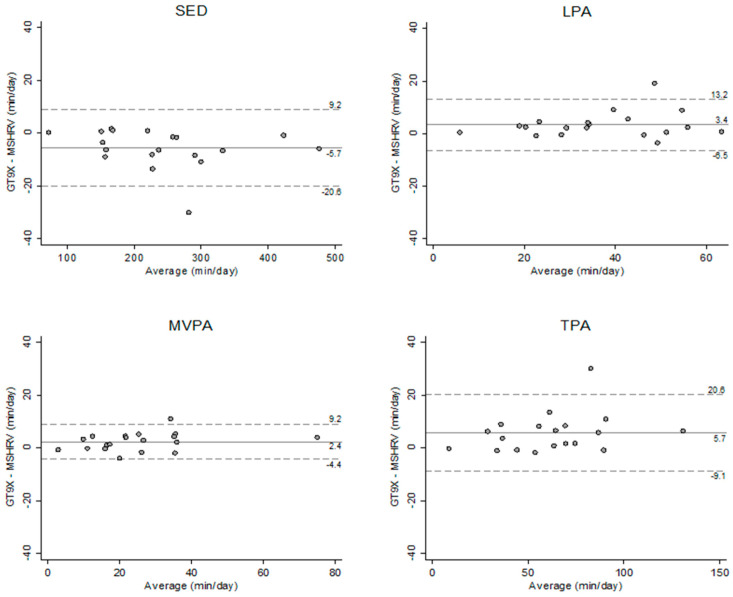
Bland–Altman plots illustrating the level of agreement in sedentary behavior and PA estimates between the GT9X and MotionSense HRV (MSHRV) under free-living conditions. Dashed lines show 95% limits of agreement (±1.96 standard deviation). SED: sedentary behavior; LPA: light PA; MVPA: moderate-to-vigorous PA; TPA: total PA.

**Figure 4:**
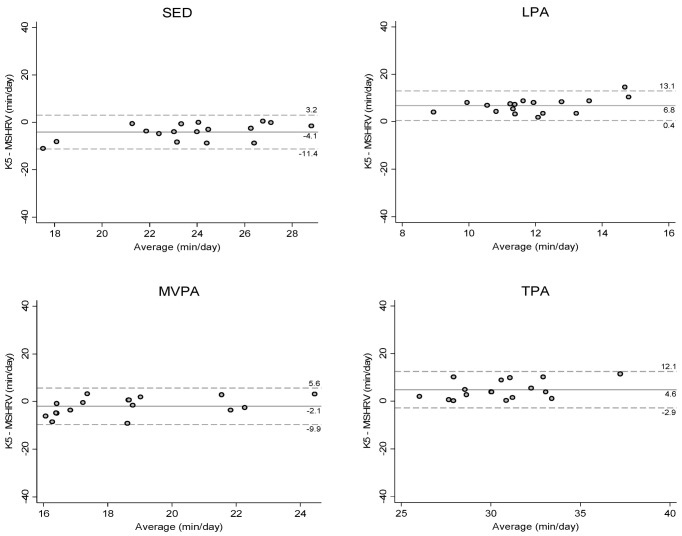
Bland–Altman plots illustrating the level of agreement in sedentary behavior and PA estimates between the Cosmed K5 and MotionSense HRV (MSHRV) under simulated free-living conditions. Dashed lines show 95% limits of agreement (±1.96 standard deviation). SED: sedentary behavior; LPA: light PA; MVPA: moderate-to-vigorous PA; TPA: total PA.

The results from the Bland–Altman plots showed that there was no apparent bias for the agreement in sedentary behavior and PA estimates between the MotionSense HRV and GT9X under free-living conditions (Figure 3); however, the results from the simulated free-living condition revealed that the MotionSense HRV tends to provide lower light PA estimates compared to the K5 (Figure 4).

The results of the equivalent tests are shown in Table 5 and Table 6. The estimates from the MotionSense HRV were equivalent to those from GT9X when the equivalence zones were set at 16.5% for sedentary behavior, 23.6% for light PA, 29% for moderate-to-vigorous PA, and 24.1% for total PA (Table 5). Under the simulated free-living conditions, the estimates from the MotionSense HRV reached equivalence when the equivalence zones of the K5 were set at 24.1% for sedentary behavior, 48.6% for light PA, 18.2% for moderate-to-vigorous PA, and 17.5% for total PA (Table 6).

## 4. Discussion

The current study evaluated the concurrent validity of the MotionSense HRV for estimating time engaged in sedentary behavior and PA against two previously established criterion measures. Our overall findings indicated that the MotionSense HRV can provide reasonably comparable measures of time spent in sedentary behavior and different PA intensities in comparison to the GT9X, which is the most widely accepted objective method for PA measurement in free-living conditions [53,54]. However, the estimates from the MotionSense HRV were less comparable when compared with those from K5 indirect calorimetry during simulated free-living conditions. More specifically, the MotionSense HRV tends to overestimate sedentary behavior and moderate-to-vigorous PA but underestimate light PA and total PA. These specific findings would be valuable to researchers and clinicians considering using the MotionSense HRV for comprehensively evaluating various health-related behaviors in a large population.

Findings in free-living conditions demonstrated that the actual mean differences (−5.7–5.7 min/day) and the mean absolute percent errors (2.4–9.1%) were relatively small between the MotionSense HRV and GT9X, suggesting that the MotionSense HRV has promising potential to accurately assess sedentary behavior and PA patterns under true free-living conditions. The mean bias and mean absolute percent error values provide a valuable indicator to determine if the MotionSense HRV accurately estimates sedentary behavior and PA patterns. Although it is challenging to directly compare our results with other studies due to the absence of published data, the observed mean absolute percent errors of the MotionSense HRV against GT9X across sedentary behavior and PA estimates can be compared with previous studies that assessed the accuracy of activity monitors compared to the ActiGraph in free-living conditions. In general, studies that used the ActiGraph as a criterion measure under free-living conditions indicated that the mean absolute percent error of 15% or less is an acceptable degree of error for sedentary behavior and PA estimates [55,56,57]. In light of the relatively low mean absolute percent error values, the present study indicated that the MotionSense HRV had a high accuracy across the activity intensity classification. Moreover, these results can be interpreted against previous validation studies that compared the outcomes between devices worn at different body locations (e.g., hip, wrist, or ankle) [54,58]. It has been reported that the accelerometer performance may vary with the device placement site [59,60,61] suggesting that the validity determined between the devices worn at different body site could be limited. Unlike those studies, our study directly compared the SED and PA estimates between the MotionSense HRV and GT9X, as both devices were placed on the same wrist and used for the same data processing algorithm using raw acceleration signals [58,62]. Due to the observed agreements for sedentary behavior and PA estimates between the MotionSense HRV and GT9X, the MotionSense HRV can be considered a valid device to measure the total spectrum of activity, including sedentary behavior and all PA intensities [63]. 

It is worth noting that the measurement errors in sedentary behavior and light PA were greater for the comparison with the indirect calorimetry than the GT9X. More specifically, the MotionSense HRV showed a large degree of underestimation for light PA relative to the K5 indirect calorimetry, indicating that light PA could be misclassified as either sedentary behavior or moderate PA. One possible explanation for the observed difference in the light PA estimate could be due to the limited number of activities prescribed at light intensity levels in the simulated free-living conditions. It is also speculated that some participants might have relatively low or high magnitudes of wrist movement when standing or performing certain activities for a short duration within light PA under the simulated free-living condition. Since the estimations of sedentary behavior and PA were based on the amount of wrist acceleration measured by the MotionSense HRV, the degree of wrist movements at the prescribed light PA might directly influence the magnitude of wrist acceleration measured by the MotionSense HRV. A previous study using the ActiGraph accelerometer demonstrated that the locomotive movements could be considered as sedentary behavior since the magnitude of wrist acceleration while standing corresponds to that of acceleration during sedentary activities in the lab-based activity protocol [48]. Accordingly, the MotionSense HRV may record low acceleration signals during locomotive activities involving limited arm movements, thus misclassifying light PA as sedentary behavior while performing the prescribed activities during the simulated free-living conditions. Likewise, the magnitude of wrist acceleration during a certain light PA with a lot of wrist movements might exceed the threshold for the light PA, resulting in the MotionSense HRV underestimating the light PA time. Consequently, our findings may suggest that the MotionSense HRV may not be an ideal device to measure activity estimates or energy expenditure for light PA in laboratory-based settings or a short period of time. Nonetheless, given that people use various arm movements even when standing in true free-living conditions, the MotionSense HRV is still an acceptable measurement tool to estimate the time spent in sedentary behavior and PA for clinical and research purposes.

In the present study, we examined the MotionSense HRV’s accuracy at both the individual and group level. Evaluating the degree of systematic error at the individual level may be more stringent because, unlike the measurement at the group level, the measurement errors may not be offset by the larger sample at the individual level [64]. The results from the Bland–Altman plots across all activity intensities showed that the mean biases in the estimates between MotionSense HRV and GT9X were small, and only one individual bias fell outside of the 95% limits of agreement. These findings suggest that the MotionSense HRV yielded relatively precise SED and PA estimates compared to the GT9X at the individual level under free-living conditions [64,65]. Moreover, we used equivalence testing to directly assess agreement between the MotionSense HRV and criterion measures at the group level [52,66]. Due to the absence of a universally acceptable equivalence zone range, the current study attempted to examine the relative equivalency, which is actual equivalence zone of the MotionSense HRV against the criterion measures, instead of determining the equivalence of the MotionSense HRV in a dichotomous manner by setting a priori a specific zone of equivalence [64]. Additionally, equivalence testing was a rigorous analytic method to assess agreement for the MotionSense HRV against the criterion measures, so it would not be used alone to define the agreement. Instead, it facilitated the evaluation of systematic error with other analytic methods, such as mean absolute present error and Bland–Altman plot. Using this approach, we were able to identify the actual equivalence regions where the 90% confidence intervals of the estimates from the MotionSense HRV completely fall within the mean estimates from the criterion measures. These findings also demonstrated that the MotionSense HRV had a relatively low measurement error at the group level in estimating sedentary behavior compared to light PA and moderate-to-vigorous PA under free-living conditions, supporting the results of other statistical analyses in this study. Thus, the findings from the equivalence testing could help researchers and clinicians to make more informed decisions on the validity of the MotionSense HRV for future use in research and in practical settings. 

With the ever-increasing interest in mobile health (mHealth), the utility of a wearable device for monitoring various physiological and behavioral factors influencing health conditions is an essential component of the mHealth system [3]. The MotionSense HRV, a wrist-worn mHealth device, is capable of measuring hand gestures and bodily movements via accelerometers and gyroscopes and interbeat intervals via PPG sensors [5]. In addition, the MotionSense HRV could provide more efficiency in data management than using multiple sensors measuring individual factors, because it is compatible with the mCerebrum mobile application that enables processing data from multiple sensors in an integrated way [5,17]. Notably, the data from the MotionSense HRV can be used to analyze activity patterns, stress, smoking events, and eating habits through specific algorithms within the mCerebrum [5,8,14,15]. Moreover, the mCerebrum mobile application supports data quality assessment and privacy management for sensor and self-report data collected by participants in both lab and field settings and wirelessly transmits the processed data to a cloud platform (called Cerebral Cortex) [5], allowing clinicians and researchers to use various biological and behavioral data just in time [3,5,17]. Therefore, the MotionSense HRV would be a useful mHealth device to accurately and comprehensively evaluate users’ various health-related behaviors along with PA patterns for patient-centered lifestyle change intervention and large-scale epidemiologic studies [17,67]. 

It is also noteworthy that the MotionSense HRV has great potential for improving its accuracy by integrating the data from other equipped sensors such as a 3-axis gyroscope and the photoplethysmogram (PPG) sensor, which can measure the angular motion, force, and orientation of the body in three dimensions and heart rate (HR) [68,69]. Indeed, a recent study reported that the combined use of gyroscope and accelerometer data in the wrist-worn ActiGraph GT9X reduced the measurement error in estimating moderate-to-vigorous PA compared with the estimates derived from accelerometry data alone [27]. This suggests that, along with the MotionSense HRV’s primary three-axis accelerometer, the use of multiple sensors together can improve the accuracy of measuring PA, as they allow one to measure very comprehensive and sophisticated bodily movements [70]. Another significant enhancement that the MotionSense HRV has over other accelerometer-only devices is the availability of a PPG sensor, which can measure HR [5,71]. Given the direct relationship between activity intensity and HR, adding HR data to the accelerometry-only algorithm has been shown to increase the device’s accuracy for estimating the amount and intensity of PA that particularly involved fewer arm movements (e.g., lower body dominant resistance exercise) [72,73]. An additional unique feature of the MotionSense HRV is GPS data capability. Compatible with smartphones, the smartphone’s GPS data can be synced with the data from the MotionSense HRV unit and then processed together via a mobile application installed on the smartphone called mCerebrum. Adding GPS data into the accelerometer-only algorithms could significantly improve the quality of the measurements, as it can incorporate rich information, including grades and locations, which particularly improve assessing PA performed outdoors. Although algorithms that incorporate all the data from multiple sensors and GPS has not been developed and thus the validity of these additional features in estimating PA was untestable in the present study, the MotionSense HRV has tremendous potential to improve its validity and usability further when the utility of its additional sensors in measuring PA is determined in subsequent research. 

This study has strengths that should be highlighted. We assessed the validity of the MotionSense HRV comprehensively in both lab-based and free-living conditions using high-quality criterion measures [42,43,44,64,74]. Moreover, we measured PA in free-living conditions for more than seven days, including at least one weekend day, in order to ensure a high reliability of PA measurement [75]. This approach was critical for determining the validity of MotionSense HRV against GT9X because the longer measurement period in this study provided more representative measures of habitual PA during free-living conditions [75,76] compared to other recent studies that determined the validity of accelerometers based on only 1–2 days of measurement [62,77]. 

This study also has limitations that are worth mentioning. Our sample consisted of Pacific Islanders and was relatively small in size, which may limit the generalizability of the findings from this study. However, the observed PA pattern in our sample (24–26 min/day of moderate-to-vigorous PA) was not significantly different from that in generally healthy populations; thus, the ethnic characteristics of our sample should not be considered as a major threat to internal validity in this study. Another limitation of this study was that basal metabolic rate (BMR) was not measured during the lab session. Although using the true BMR rather than resting metabolic rate is more desirable for estimating MET values, the BMR test requires a significant participant burden (i.e., 12-hour fasting) as well as exclusive lab settings (i.e., sleep lab), which was not feasible for the present study. Lastly, this study could not measure aquatic activities performed during free-living conditions because the MotionSense HRV is not completely waterproof. 

## 5. Conclusions

The MotionSense HRV can provide reasonably valid sedentary behavior and PA estimates in relation to the GT9X in free-living conditions. Relative to the K5 indirect calorimetry, however, we found that the MotionSense HRV had sizable measurement errors for sedentary behavior in lab-based and/or short-term research. Considering the accuracy of MotionSense HRV’s sedentary behavior and PA estimates under free-living conditions, we confirmed the promising potential of using MotionSense HRV alone to monitor a variety of health-related behaviors, including PA patterns, stress response, smoking events, and eating habits, for research and clinical purposes. Accordingly, this study suggests that the developers of the MotionSense HRV should consider adding specific algorithms that measure the user’s daily sedentary behavior and PA patterns using the internal sensors of the MotionSense HRV.

## Figures and Tables

**Table 1:** Description of activities by intensity.

Intensity Type	Activity	Duration
Sedentary Behavior	Resting in the supine position	5 min
	Watching TV in the sitting position	5 min
	Reading books in the sitting position	5 min
	Typing computer in the sitting position	5 min
Transit #1		1 min
Light Physical Activity	Fidgeting in the standing position	5 min
	Walking at a casual pace (1–1.5 mph)	5 min
	Housekeeping/work (i.e., setting the table)	5 min
	Exploring/sorting (i.e., stacking light boxes)	5 min
Transit #2		1 min
Moderate-to-Vigorous Physical Activity	Walking briskly (2.5–3.0 mph)	5 min
	Running at a moderate pace (3.5–4.0 mph)	5 min
	Running at a fast pace (4.5–5.0 mph)	5 min
	Full body free play (e.g., throwing ball, basketball, soccer, tennis, jumping jack)	5 min

**Table 2:** Participant characteristics by gender, mean ± standard deviation.

	All (N = 20)	Male (N = 8)	Female (N = 12)	*p*-Value *
Age (years)	32.5 ± 15.1	29.5 ± 13.1	34.5 ± 16.5	0.48
Height (cm)	172.0 ± 6.9	178.8 ± 2.5	167.5 ± 4.6	<0.01 **
Weight (kg)	90.1 ± 12.5	96.1 ± 13.0	86.1 ± 10.9	0.08
Waist (cm)	97.7 ± 10.9	98.1 ± 13.5	97.5 ± 9.4	0.91
BMI (kg/m^2^)	30.5 ± 4.0	30.1 ± 4.3	30.7 ± 4.0	0.74
Weight Status (%)				
Normal	15%	12.5%	17%	0.78
Overweight/obese	75%	87.5%	83%	0.78
Wear time (min/day)	297.7 ± 119.7	318.3 ± 158.9	283.9 ± 90.3	0.54

* *p*-value for gender difference; ** *p* < 0.05.

**Table 3:** Mean differences (SE) and Mean Absolute Percent Errors of Sedentary behavior and Physical activity (PA) between the GT9X and MotionSense HRV under free-living conditions.

Intensity	GT9X (SD)	MotionSense HRV (SD)	Mean diff. (SE)	MAPE (%)
SED	237.1 min (97.8)	242.8 min (99.5)	−5.7 min (1.7)	2.4%
LPA	38.6 min (15.8)	35.3 min (14.8)	3.3 min (1.1)	8.7%
MVPA	26.4 min (16.2)	24.0 min (15.1)	2.4 min (0.8)	9.1%
TPA	65.0 min (29.3)	59.2 min (27.2)	5.7 min (1.7)	8.8%

MAPE: Mean Absolute Percent Error; SD: standard deviation; SE: Standard Error; SED: sedentary behavior; LPA: light PA; MVPA: moderate-to-vigorous PA; TPA: total PA.

**Table 4:** Mean differences (SE) and Mean Absolute Percent Errors of Sedentary behavior and Physical activity (PA) between the Cosmed K5 and MotionSense HRV under simulated free-living condition.

Intensity	Cosmed K5 (SD)	MotionSense HRV (SD)	Mean diff. (SE)	MAPE (%)
SED	21.6 min (4.2)	25.7 min (2.6)	−4.1 min (0.9)	18.9%
LPA	15.3 min (2.7)	8.5 min (1.6)	6.8 min (0.8)	44.2%
MVPA	17.5 min (3.8)	19.6 min (2.4)	−2.1 min (0.9)	12.3%
TPA	32.8 min (3.9)	28.2 min (2.6)	4.6 min (0.9)	14.1%

MAPE: Mean Absolute Percent Error; SD: standard deviation; SE: Standard Error; SED: sedentary behavior; LPA: light PA; MVPA: moderate-to-vigorous PA; TPA: total PA.

**Table 5:** 90% Confidence Intervals (CIs) from the MotionSense HRV and Equivalence Zones (EZs) from the GT9X in free-living conditions.

Intensity	GT9X (SE)	MotionSense HRV (SE)	90% CI of MotionSense HRV	EZ of GT9X	EZ (%)
SED	237.1 min (4.2)	242.8 min (22.4)	198.18 to 275.7 min/d	197.98 to 276.2 min/d	16.5%
LPA	38.6 min (3.6)	35.3 min (3.2)	29.51 to 40.7 min/d	29.49 to 47.71 min/d	23.6%
MVPA	26.4 min (3.7)	24.0 min (3.3)	18.74 to 30.28 min/d	18.72 to 34.01 min/d	29.0%
TPA	65.0 min (6.7)	59.2 min (5.9)	49.35 to 69.87 min/d	49.31 to 80.62 min/d	24.1%

SE: standard error; SED: sedentary behavior; LPA: light PA; MVPA: moderate-to-vigorous PA; TPA: total PA.

**Table 6:** 90% Confidence Intervals (CIs) from the MotionSense HRV and Equivalence Zones (EZs) from the K5 indirect calorimetry in simulated free-living conditions.

Intensity	Cosmed K5 (SE)	MotionSense HRV (SE)	90% CI of MotionSense HRV	EZ of Cosmed K5	EZ (%)
SED	21.6 min (4.2)	25.7 min (2.6)	24.64 to 26.85 min/d	16.43 to 26.86 min/d	24.1%
LPA	15.3 min (2.7)	8.5 min (1.6)	7.88 to 9.2 min/d	7.86 to 22.73 min/d	48.6%
MVPA	17.5 min (3.8)	19.6 min (2.4)	18.60 to 20.63 min/d	14.29 to 20.65 min/d	18.2%
TPA	32.8 min (3.9)	28.2 min (2.6)	27.06 to 29.25 min/d	27.03 to 38.5 min/d	17.5%

SE: standard error; SED: sedentary behavior; LPA: light PA; MVPA: moderate-to-vigorous PA; TPA: total PA.

## Data Availability

The datasets of the current study are available from the authors on reasonable request.

## Supplementary Materials

The following are available online at https://www.mdpi.com/1424-8220/21/4/1411/s1. Table S1: evaluation of the usability of the MotionSense HRV, mean ± standard deviation or percent.
