# Supplementary material for: The Validity of MotionSense HRV in Estimating Sedentary Behavior and Physical Activity under Free-Living and Simulated Activity Settings
Source: Sensors (Basel). 2021 Feb 18;21(4):1411. doi: 10.3390/s21041411 (PMC7922785; doi:10.3390/s21041411)
Supplement: Supplementary file 1 [file sensors-21-01411-s001.pdf]

Table S1. Evaluation of the Usability of MotionSense HRV, Mean ± Standard deviation or Percent

Questions	Score Range	Response Rate (%)	Response Score
How burdensome was it to carry the smartphone and devices during the past 7 days?	†1 = Not at all, 5 = Extremely	100	3.0 ± 1.3
What is your overall experience on the mobile monitoring system?	‡1 = Poor, 5 =Excellent	100	3.2 ± 1.0
How do you like the phone that we gave you?	†1 = Not at all, 5 = Extremely	100	3.6 ± 0.9
Do you think the software on the phone is easy to use?	†1 = Not at all, 5 = Extremely	100	4.2 ± 1.0
Was the instruction page useful for you to use the mobile system?	†1 = Not at all, 5 = Extremely	100	4.2 ± 0.8
During the consenting procedure, how well did the investigator explain the project to you?	‡1 = Poor, 5 =Excellent	100	4.7 ± 0.6
Considering the privacy-protection procedures, are you concerned about the privacy issue?	1 = Yes, 2 = No	100	*10%
Did you get quick feedback whenever you contact our people?	1 = Yes, 2 = No	100	*100%
If you received feedback, was that useful for you to solve the problem?	1 = Yes, 2 = No	100	*100%
Based on your experience of the last 10 days, do you think you will still participate in similar studies that require you to carry a smartphone and wristbands?	1 = Yes, 2 = No	100	*75%
Additional feedback from participants	- I would suggest if possible fewer devices because it was hard to keep track of all of them/charge all of them each day.<br>- Waterproof?<br>- Consolidate equipment. Maybe just wristbands or just watch + pedometer.<br>- A higher distance from the phone would be great too.<br>- Self-recording monitors that don't require a separate smartphone (or download an app on my own device).<br>- More comfortable straps (buckle hurt skin after a while). Possible ankle location rather than wrist?		

†1: Not at all; 2: A little bit; 3: Moderately; 4: Very; 5: Extremely
‡1: Poor; 2: Fair; 3: Good; 4: Very good; 5: Excellent
*Percent of participants who responded 'Yes.'
